# The impact of different preceding crops on soil nitrogen structure and nitrogen cycling in tobacco-planting soil

**DOI:** 10.1038/s41598-024-52285-z

**Published:** 2024-01-20

**Authors:** Ming Liu, Rujun Xue, Ningbo Han, Shanqin Yang, Dexun Wang, Yanxia Hu, Kaiyuan Gu, Jiaen Su

**Affiliations:** 1 Dali Prefecture Branch of Yunnan Tobacco Company, Dali, 671000 Yunnan China; 2 Weishan City Branch of Yunnan Tobacco Company, Weishan, 672400 Yunnan China

**Keywords:** Plant sciences, Solid Earth sciences

## Abstract

Soil nitrogen content, structure, and nitrogen cycling play a crucial role in tobacco growth quality, with different preceding crops having varying impacts on tobacco cultivation soil. This study conducted using field experiments, employed three treatments with different preceding crops, namely tobacco, barley, and rapeseed, to investigate the effects of different preceding crops on soil nitrogen structure and the expression levels of soil nitrogen cycling-related functional genes in tobacco cultivation soil. The results indicated that different preceding crops had varying effects on the content of different nitrogen forms in tobacco cultivation soil. Ammonium nitrogen and nitrate nitrogen were the two nitrogen forms which were most influenced by preceding crops, with the ammonium nitrogen content in soils following barley and rapeseed preceding crops increasing by 82.88% and 63.56%, respectively, compared to sole tobacco cultivation. The nitrate nitrogen content in tobacco cultivation soil was 26.97% higher following barley preceding crops and 24.39% higher following rapeseed preceding crops compared to sole tobacco cultivation. Simultaneously, different preceding crops also affected the expression levels of nitrogen cycling-related genes in tobacco cultivation soil. In the nitrification process, *amoA* was significantly impacted, with its expression reduced by 64.39% and 72.24% following barley and rapeseed preceding crops, respectively, compared to sole tobacco cultivation. In the denitrification process, except for the *narG* gene, all other genes were subjected to varying degrees of inhibition when preceded by barley and rapeseed crops. Correlation analysis between soil nitrogen structure and the expression levels of nitrogen cycling-related genes revealed that increased nitrogen levels suppressed the expression of *Arch-amoA*. Additionally, ammonium nitrogen strongly influenced the expression levels of most soil nitrogen cycling functional genes. In conclusion, preceding crops alter soil nitrogen structure, possibly due to changes in soil microorganisms, and different preceding crops modified the expression levels of nitrogen cycling-related genes in tobacco cultivation soil, consequently affecting the proportions of various nitrogen forms in the soil.

## Introduction

The soil nitrogen content plays a crucial role in influencing the growth and yield of tobacco plants, as nitrogen is identified as a key limiting factor in tobacco growth^1^. Plants require nitrogen elements to support processes such as protein synthesis, chlorophyll formation, and the synthesis of other biological molecules, all of which are vital for the normal growth and development of plants. Adequate nitrogen supply contributes to promoting leaves growth and roots development, thereby maintaining the overall health of the plant^2^. Furthermore, an appropriate level of soil nitrogen content can also enhance tobacco yield and leaf quality. Under conditions of sufficient nitrogen, tobacco plants can more efficiently carry out photosynthesis, absorb and utilize more nutrients, leading to an increase in leaf number and size, consequently enhancing total yield and product quality^3^. In addition, fluctuations in soil nitrogen levels directly impact the chemical composition of tobacco^4^. Typically, higher nitrogen supply in the soil can lead to a reduction in nicotine content in tobacco, while lower nitrogen supply may result in increased nicotine content^5^. These changes in chemical composition have significant implications for the quality and purposes of tobacco products.

Tobacco, as a member of the Solanaceae family, is typically cultivated in crop rotations with other crops. Different preceding crops, owing to their distinct growth and developmental characteristics, exert various effects on the tobacco soil. Research had shown that certain plants, such as legumes and grasses, possess higher biomass production and root residue, which can significantly enhance soil organic matter content^6–8^. Zhou et al. conducted research on four different planting methods, including winter milk vetch, winter rape, winter garlic, and a winter rotation intensification with potato, milk vetch, and rape^9^. They found that the effects of crop rotation on rice yield and soil carbon–nitrogen content varied among different crops. Similarly, Mesfin et al., demonstrated in their study that the rotation of faba bean, field pea, and lentil with wheat influenced wheat's nitrogen uptake efficiency and altered soil nitrogen structure^10^. Preceding crops can also impact soil enzyme activities, particularly those related to nitrogen^11^. Different preceding crops leave behind various types and quantities of root residues in the soil, which influence soil microbial community diversity, richness, and enzyme activity^12^. Study have revealed that certain preceding crops, such as legumes, can increase soil urease and nitrification enzyme activities, facilitating nitrogen conversion and provision^13^. In contrast, other crops may reduce soil enzyme activity, thereby limiting nitrogen supply. Various preceding crops also affect the distribution and forms of nitrogen in the soil^14,15^. Research indicates that some preceding crops can promote the accumulation of nitrogen in the soil, particularly in organic forms such as organic nitrogen and ammonium nitrogen^16^. These nitrogen forms are more favorable for tobacco growth because they not only provide nitrogen nutrition but also reduce competition from nitrate nitrogen for tobacco. Therefore, selecting appropriate preceding crops can improve the distribution of nitrogen forms in the soil, contributing to increased tobacco yield and quality.

Soil nitrogen cycling is an essential component of soil microbial substance cycling and energy transformation, involving a variety of complex and large microorganisms^17^. Among them, nitrogen fixation and nitrification–denitrification processes are crucial components of soil nitrogen cycling. Biological Nitrogen Fixation (BNF) is a specific physiological function of nitrogen-fixing microorganisms, where they reduce atmospheric nitrogen gas (N_2_) to ammonia nitrogen (NH_3_) through their nitrogenase enzymes^18^. Research has found that a class of *nif* genes is responsible for carrying out nitrogen fixation in these microorganisms. *nifH* is one member of these nitrogen-fixing genes, and its gene sequence is not highly conserved, exhibiting structural variations among different bacterial strains. Therefore, the *nifH* gene can be employed to study the diversity of nitrogen-fixing bacterial groups in soil, essentially serving as a tool for species identification^19^. On the other hand, nitrification and denitrification processes involve multiple bacteria and soil enzymes. In studies of ammonia oxidation, molecular-level analysis typically targets the *amoA* gene encoding the AMO subunit. Soil nitrification refers to the process of NH_4_^+^ generating NO_3_^−^ under the action of nitrifying microorganisms in soil, which is actually the oxidation of NH_4_^+^ in Ammonia-Oxidizing Bacteria (AOB) and Ammonia-Oxidizing Archaea (AOA) are oxidized to nitrite NO_2_^−^ and nitrate NO_3_^−^, which is an important part of measuring the efficiency of the entire nitrogen cycle. It was found that AOB and AOA participated in and led the nitrification process depending on their Ammonia Monooxygenase (AMO). The gene fragment compiling AMO was mainly composed of three subunits, AMO-A, AMO-B and AMO-C. The corresponding genes are *amoA*, *amoB* and *amoC*^20^. Two functional genes, *amoA* and *amoB*, are mainly responsible for coding in the soil environment^21^. Among them, *amoA* gene encoding AMO-A subunit can independently express and synthesize AMO to complete the enzymatic reaction to ensure the smooth nitrification process. Moreover, *amoA* gene is generally present in AOA and AOB, and has higher conserved and expressed ability than *amoB*. Therefore, *amoA* gene is considered to be a typical functional gene in the study of nitrifying microorganisms in soil, and is widely used to analyze the community composition of AOA and AOB^22^. Cultivation experiments conducted by Yu et al., and others have revealed that a relatively low level of dissolved oxygen can promote the transcription of *amoA* in ammonia-oxidizing bacteria (AOB)^23^. Research by Bollmann et al., and colleagues showed that even after short-term ammonia starvation treatment of Nitrosospira, the mRNA of the *amoA* gene in Nitrosospira remained present, and ammonia oxidation activity remained at a high level^24^. This indicates that the *amoA* subunit exhibits both strong adaptability and stability. In the denitrification process, bacteria reduce nitrogen compounds in nitrate (NO_3_^−^) to nitrogen gas (N_2_) through a series of intermediate products (NO_2_^−^, NO, N_2_O, etc.). Various enzymes, including nitrate reductase, nitrite reductase, nitric oxide reductase, and nitrous oxide reductase, participate in this process. Genes such as *narG, narZ*, and *nirK*, which encode these enzymes, can reflect the level of nitrification activity in the soil.

We postulate that different preceding crops in Yunnan, China, are likely to induce changes in the nitrogen composition of tobacco-growing soils. We speculate that such alterations may be a contributing factor to modifications in soil nitrogen cycling. It is conceivable that a strong correlation exists between the nitrogen structure of tobacco-growing soils and the nitrogen cycling processes occurring within them.

## Materials and methods

### Experiment design

This experiment was conducted in December 2022 in Weishan County, Dali Autonomous Prefecture, Yunnan Province, China (E 100.30, N 25.23, altitude 2000 m). The main physical and chemical properties of the soil were as follows: soil bulk density: 1.21 g cm^−3^, pH value: 6.47, organic matter content: 28.00 g kg^−1^, total nitrogen content: 1.68 g kg^−1^, total phosphorus content: 1.46 g kg^−1^, total potassium content: 34.54 g kg^−1^, available phosphorus: 18.13 mg kg^−1^, available potassium: 270.23 mg kg^−1^, and alkaline hydrolyzable nitrogen: 35.23 mg kg^−1^.

The field trial employed a randomized block design with three treatments, each with three replicates. Each replicate plot covered an area of 100 m^2^. All preceding crops in the plots were tobacco, with the cultivar being Hongda. The three treatments were as follows: (1) A: No cultivation of any crops; (2) B: Cultivation of barley (cultivar Kunlun 15); (3) C: Cultivation of rapeseed (cultivar Huayou 5). Both Kunlun 15 barley and Huayou 5 rapeseed are widely used local varieties. Barley and rapeseed were sown in December 2022. Row spacing for rapeseed was 25 cm with a plant spacing of 20 cm, while row spacing for barley was 25 cm with a plant spacing of 10 cm. Basal fertilizers were applied before planting, consisting of urea and compound fertilizer. Urea was applied at a rate of 10 kg/acre, and compound fertilizer (15:15:15) was applied at a rate of 12.5 kg/acre. Additional fertilization was carried out in February 2023, with urea applied at a rate of 2.5 kg/acre for both barley and rapeseed. Treatment A involved no crop cultivation and no fertilizer application. All other field management practices followed local field management standards, and harvest was conducted in May 2023.

### Soil sample collection and determination

In this experiment, soil sample collection was carried out after the harvest of crops in 2023. For each treatment, ten residual crop remnants were selected after harvesting. The roots were excavated, and adhering soil on the root surfaces was shaken off. A gentle brushing was used to remove and collect the rhizosphere soil still adhering to the roots. A portion of this soil was immediately preserved in liquid nitrogen for the determination of soil nitrogen cycling-related gene expression levels. Another portion was placed in a cool, shaded area for air drying to be used in determining different forms of nitrogen in the soil.

The experiment involved the measurement of four nitrogen forms in the soil, with the method of Liu^25^: total nitrogen (TN), soluble nitrogen (STN), ammonium nitrogen (AMN), and nitrate nitrogen (NIN). In addition, four nitrogen forms were calculated: inorganic nitrogen (IN), organic nitrogen (ON), soluble organic nitrogen (SON), and insoluble organic nitrogen (ION). Total nitrogen in the soil was determined using the continuous flow method (LY/T1228-2015), soluble nitrogen was measured using the alkaline diffusion method (LY/T1228-2015), while ammonium nitrogen and nitrate nitrogen were determined using the continuous flow method (LY/T1228-2015). Inorganic nitrogen = ammonium nitrogen + nitrate nitrogen; organic nitrogen = total nitrogen − inorganic nitrogen; soluble organic nitrogen = soluble nitrogen − inorganic nitrogen, and insoluble organic nitrogen = organic nitrogen − soluble organic nitrogen.

### DNA extraction and quantification

DNA was extracted from 0.5 g soil using the Fast DNA Spin Kit for Soil (MP Biomedicals, CA, USA) and then purified using PowerClean DNA Clean-up Kit (Mobio, CA, USA) according to the manufacturers' protocols. The quality and concentration of the extracted DNA was evaluated by gel electrophoresis (0.8% agarose) and NanoDrop spectrophotometer (NanoDrop Technologies, Wilmington, USA), and the extracted DNA was subsequently stored at − 20 °C. Functional genes of soil nitrogen cycling (*amoA, arch-amoA, narG, nirK, nirS, norB, nosZ, and nifH*) were quantified using q-PCR in a CFX96 Optical Real-Time Detection System (Bio-Rad Laboratories Inc., Hercules, CA, USA). Each reaction in 25 μL contained the specific primer set for each group: *amoA*-F/*amoA*-R (5ʹ-GGG GTT TCT ACT GGT GGT-3ʹ/5ʹ-CCC CTC KGS AAA GCC TTC TTC-3ʹ); *Arch-amoA*-F/*Arch-amoA*-R(5ʹ-TAA TGG TCT GGC TTA GAC G-3ʹ/5ʹ-CGG CCA TCC ATC TGT ATG T-3ʹ); *norG*-F/*norG*-R (5ʹ-CTC GAY CTG GTG GTY GA-3ʹ/5ʹ-TTY TCG TAC CAG GTS GC-3ʹ); *nirK*-F/*nirK*-R (5ʹ-GGM ATG GTK CCS TGG CA-3ʹ/5ʹ-GCC TCG ATC AGR TTR TGG-3ʹ); *nirS*-F/*nirS*-R (5ʹ-CCT AYT GGC CGC CRC ART-3ʹ/5ʹ-CGT TGA ACT TRC CGG T-3ʹ); *norB*-F/*norB*-R (5ʹ-GGT GGT CGA GAA GTG GCT CT-3ʹ/5ʹ-GAC CTC AAG GGT GGA GAA C-3ʹ); *nosZ*-F/*nosZ*-R (5ʹ-WCS YTG TTC MTC GAC AGC CAG-3ʹ/5ʹ-CAK RTG CAK SGC RTG GCA GAA-3ʹ); *nifH*-F/*nifH*-R (5ʹ-AAA GGY GGW ATC GGY AAR TCC ACC AC-3ʹ/5ʹ-TTG TTS GCS GCR TAC ATS GCC ATC AT-3ʹ); 515F/907R (5ʹ-GTG CCA GCM GCC GCG G-3ʹ/5ʹ-CCG TCA ATT CMT TTR AGT TT-3ʹ). Each reaction mixture (25 μL) consisted of 12.5 μL 1 × SYBR Premix Ex Taq (Takara, Tokyo, Japan), 0.25 μL of each primer, and 1 μL of DNA template that contained approximately 1–10 ng of DNA. A negative control was run with sterilized distilled water. Amplification was initiated by denaturation at 95 °C for 3 min, followed by 35 cycles of denaturation at 95 °C for 10 s, annealing at 55 °C for 30 s, extension at 72 °C for 30 s, and the plate was read at 80 °C. Melting curves and agarose gel electrophoresis were used to examine the specificity of the amplified products.

### Statistical analysis

Data were statistically analyzed using the SPSS 19.0 software package (IBM, Armonk, USA) for Windows. Differences in all the data were assessed by one-way ANOVA followed by the Tukey’s HSD test at *P* ≤ 0.05. The relationships between genes abundance and soil nitrogen structure were subjected to the Redundancy Discriminant Analyses (RDA). Data were normalized when needed to meet the analysis assumptions. R software (Version 4.0.0) was employed to perform the following analyses. Correlation among the genes abundance and soil nitrogen structure was calculated using principal coordinate analysis (PCoA) in the “vegan” package. The aggregated boosted tree analysis (ABT) using the “gbm” package with 5000 trees for boosting, tenfold cross-validation, and three-way interactions.

## Results

### Effects of different former crops on different forms of nitrogen in tobacco-growing soil

**Figure 1:**
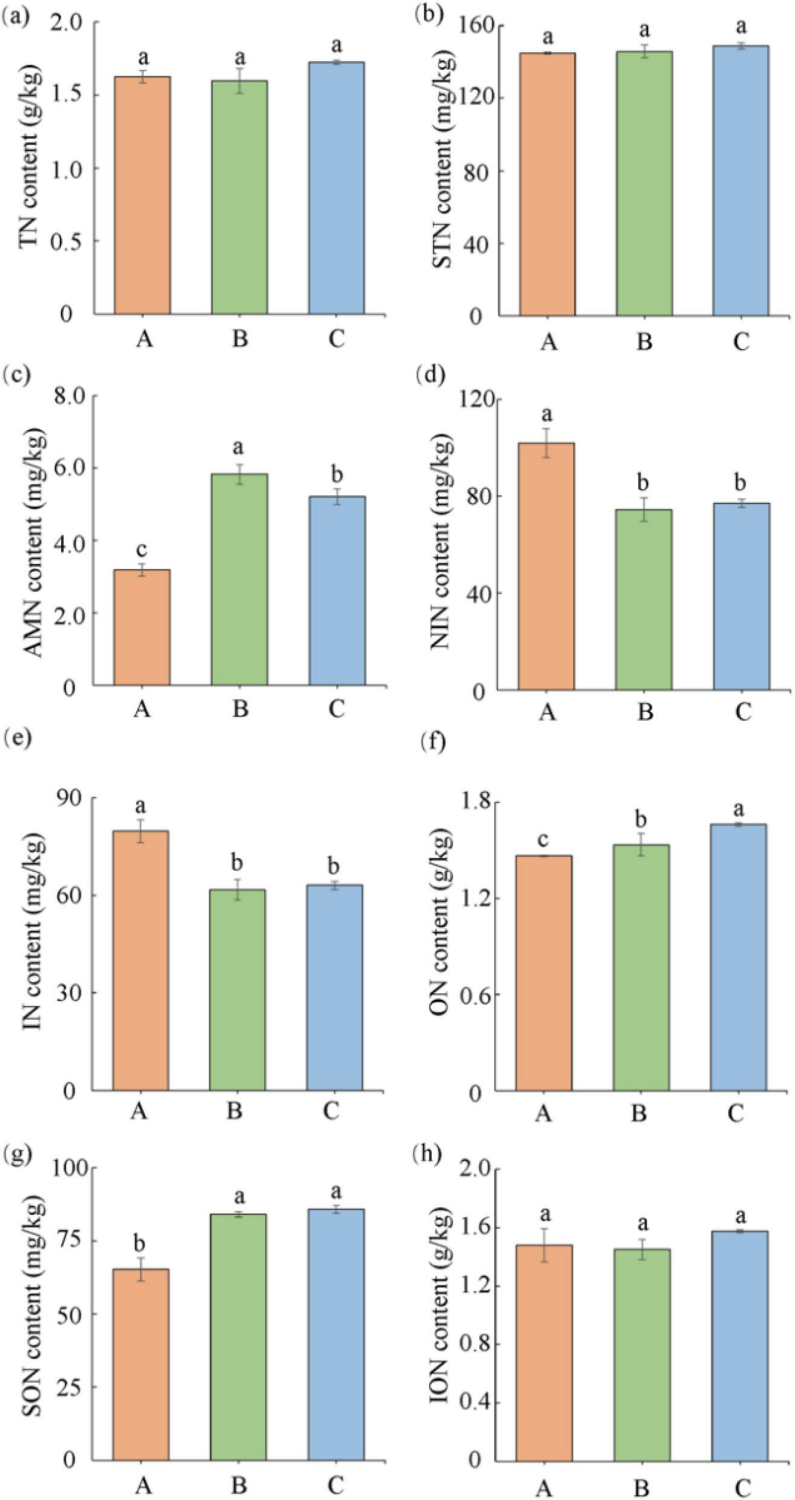
Soil nitrogen content in different forms under different treatments. Total nitrogen (**a**), soluble nitrogen (**b**), ammonium nitrogen (**c**), nitrate nitrogen (**d**), inorganic nitrogen (**e**), organic nitrogen (**f**), soluble organic nitrogen (**g**), and insoluble organic nitrogen (**h**). Different lower letters indicate significant differences between processes (p < 0.05).

**Figure 2:**
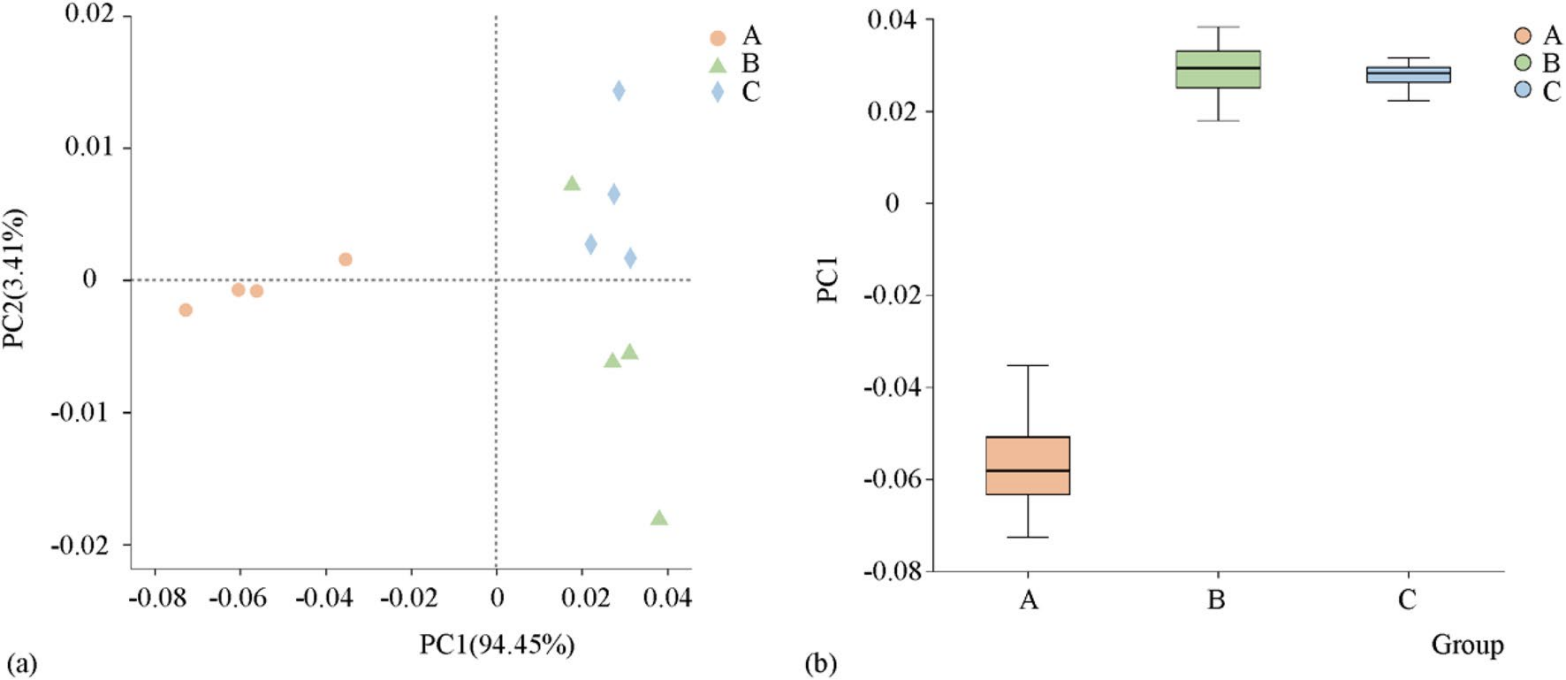
PCoA analysis and box diagram of soil nitrogen structure.

The impact of different preceding crops on the nitrogen content in various forms in tobacco-growing soils varies (Fig. 1). However, when data were subjected to dimensionality reduction (Fig. 2), the results showed that A was different from other treatments, while B and C was similarity. Regarding ammonium nitrogen (AMN), there were significant differences among all three treatments, with B > C > A. The AMN content in Treatment B was 82.88% higher than in Treatment A; in Treatment C, it was 63.56% higher than in Treatment A. For nitrate nitrogen (NIN), Treatment A had significantly higher NIN content than Treatments B and C, while there was no significant difference between Treatments B and C. The NIN content in Treatment A was 26.97% higher than in Treatment B and 24.39% higher than in Treatment C. Similarly to NIN, the inorganic nitrogen (IN) content in Treatment A was significantly higher than in Treatments B and C, with no significant difference between Treatments B and C. The IN content in Treatment A was 22.58% higher than in Treatment B and 20.87% higher than in Treatment C. In contrast to IN, the organic nitrogen (ON) content in tobacco-growing soils differed significantly among all treatments. Treatment C had 13.45% more ON than Treatment A and 8.29% more ON than Treatment B. However, for soluble organic nitrogen (SON), the SON content in tobacco-growing soils under Treatment A was significantly lower than in Treatments B and C. It was 22.36% less than in Treatment B and 23.93% less than in Treatment C. Meanwhile, there was no significant difference in SON content between Treatments B and C. For total nitrogen (TN), soil total nitrogen (STN), and inorganic nitrogen (ION), there were no significant differences in their content among tobacco-growing soils under Treatments A, B, and C.

### Effects of different former crops on the expression levels of nitrogen cycling-related genes in tobacco-planting soil

**Figure 3:**
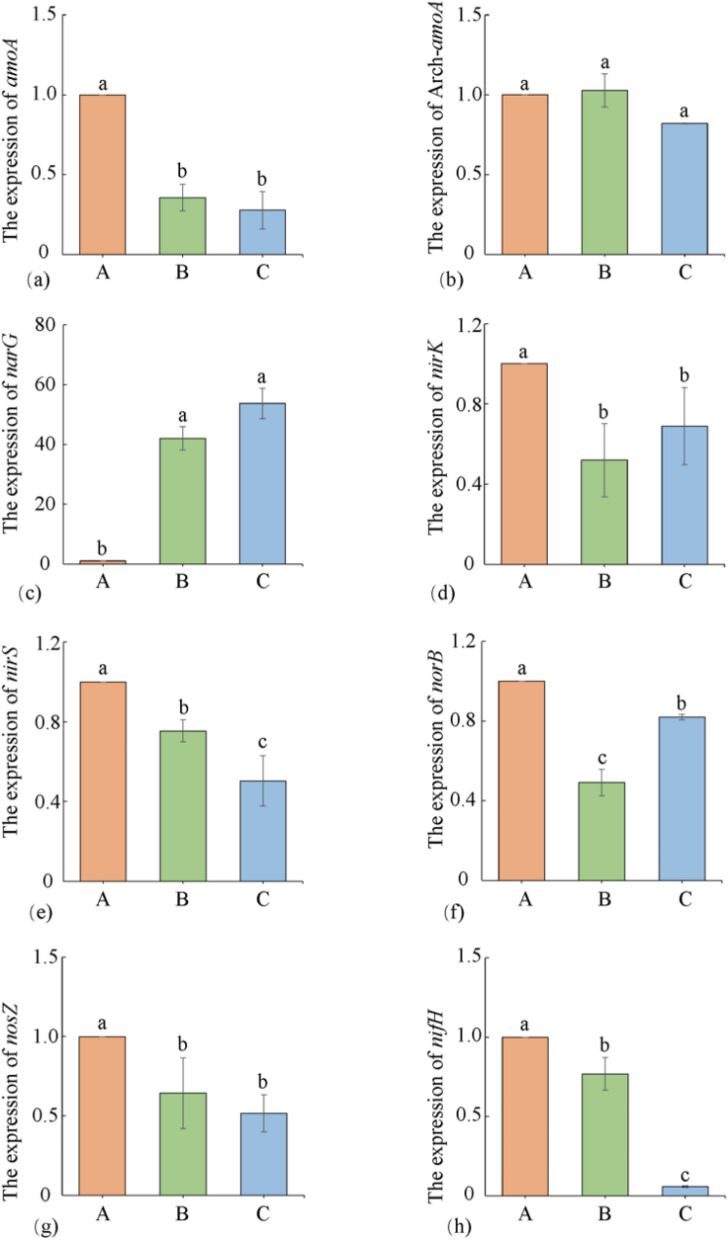
Soil nitrogen cycling-related genes expression under different treatments. *amoA* (**a**), *Arch-amoA* (**b**), *narG* (**c**), *nirK* (**d**), *nirS* (**e**), *norB* (**f**), *nosZ* (**g**), and *nifH* (**h**). Different lower letters indicate significant differences between processes (p < 0.05).

In the soil nitrogen cycling process, nitrification and denitrification are two crucial components. Figure 3 illustrated the expression levels of key genes related to soil nitrogen cycling (nitrification, denitrification, and nitrogen fixation) in tobacco-growing soil with different preceding crops. In the nitrification process, *amoA* was significantly affected, and the expression of the *amoA* gene inhibited in Treatments B and C. Under Treatment B, the *amoA* expression decreased by 64.39% compared to Treatment A, and under Treatment C, the *amoA* expression decreased by 72.24% compared to Treatment A. Conversely, the expression of *Arch-amoA* in tobacco-growing soil was minimally affected by the treatments.In the denitrification process, except for the *narG* gene, all other genes were inhibited to varying degrees in Treatments B and C. Under Treatments B and C, the expression levels of the *narG* gene in tobacco-growing soil were 42.01 times and 53.73 times higher than in Treatment A, respectively, indicating that Treatments B and C significantly promoted the expression of the *narG* gene. Meanwhile, in Treatments B and C, the *nirK, nirS, norB, and nosZ* genes were all inhibited to different extents. The expression of the *nirK* gene was 48.06% lower in Treatment B and 18.04% lower in Treatment C compared to Treatment A. The *nirS* gene showed 24.55% lower expression in Treatment B and 49.60% lower expression in Treatment C compared to Treatment A. The *norB* gene exhibited 50.83% lower expression in Treatment B and 18.04% lower expression in Treatment C compared to Treatment A. The *nosZ* gene displayed 35.63% lower expression in Treatment B and 48.42% lower expression in Treatment C compared to Treatment A. The expression level of the *nifH* gene reflected the diversity of nitrogen-fixing bacteria in the soil. In Treatments B and C, the expression of the *nifH* gene was significantly lower than in Treatment A. Specifically, in Treatment B the *nifH* gene expression was 23.14% lower than that in Treatment A, and it was nearly absent in Treatment C, with a remarkable 94.27% decrease compared to Treatment A.

### Effects of soil nitrogen structure on expression levels of soil nitrogen cycling-related genes

**Figure 4:**
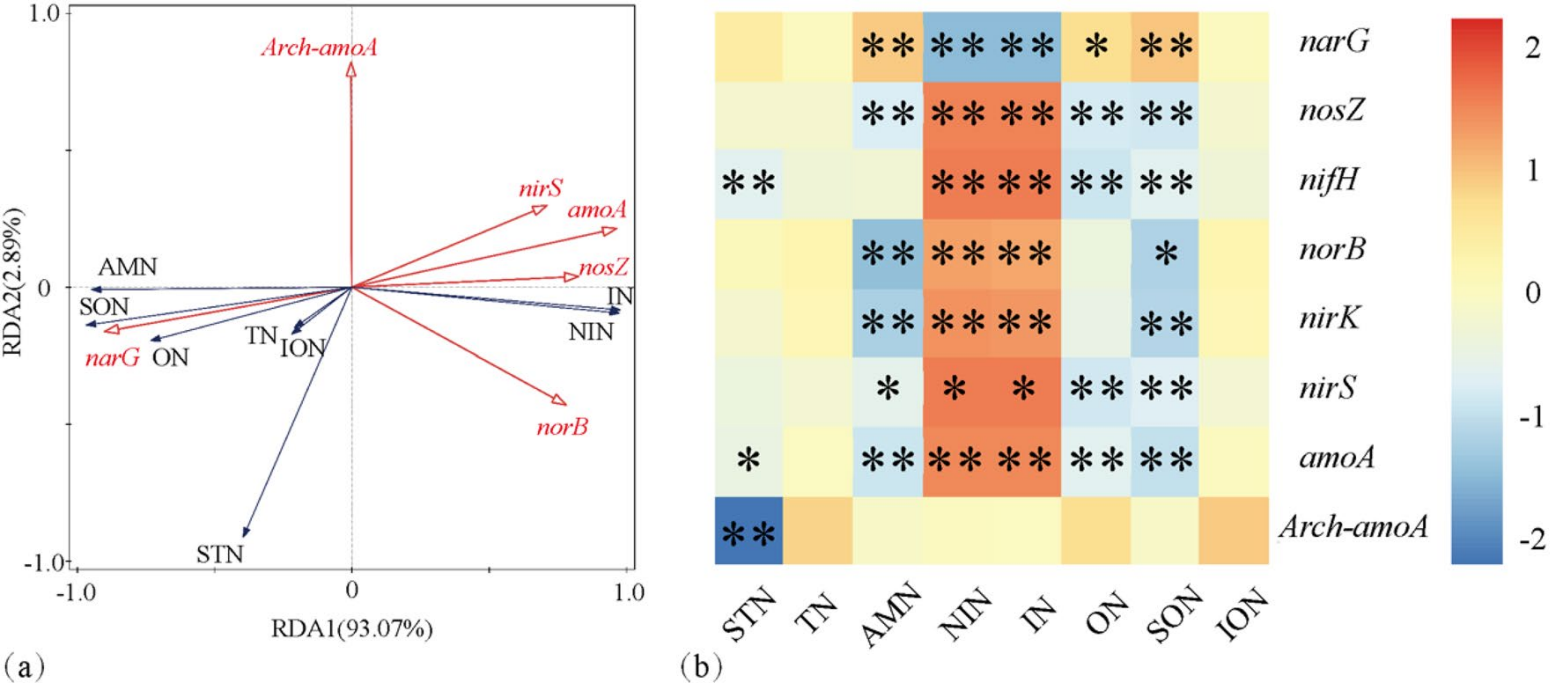
Effect of soil nitrogen structure on soil gene expression. ^*^Significant at the 0.05 probability level. **Significant at the 0.01 probability level.

A correlation analysis was conducted between different forms of nitrogen in tobacco-growing soil and the expression levels of soil nitrogen cycling-related genes (Fig. 4). It was observed that *Arch-amoA* showed no significant correlations with most forms of nitrogen but exhibited a significant negative correlation with STN. This indicated that an increase in nitrogen content, as an environmental factor, inhibited the expression of *Arch-amoA.* On the other hand, different forms of nitrogen had a significant impact on the expression of other genes. Particularly, IN and NIN significantly influenced the expression of nitrogen cycling-related genes, except for *narG*, with which they exhibited a negative correlation. These forms of nitrogen were significantly positively correlated with other genes, including *amoA, nirK, nirS, norB, nosZ*, and *nifH*. For *amoA, nirK, nirS, norB, nosZ*, and *nifH*, only IN and NIN were positively correlated with gene expression, while other forms of nitrogen exhibited negative correlations or showed no significant relationships. Notably, *narG* exhibited a positive correlation with ON, SON, and AMN.

**Figure 5:**
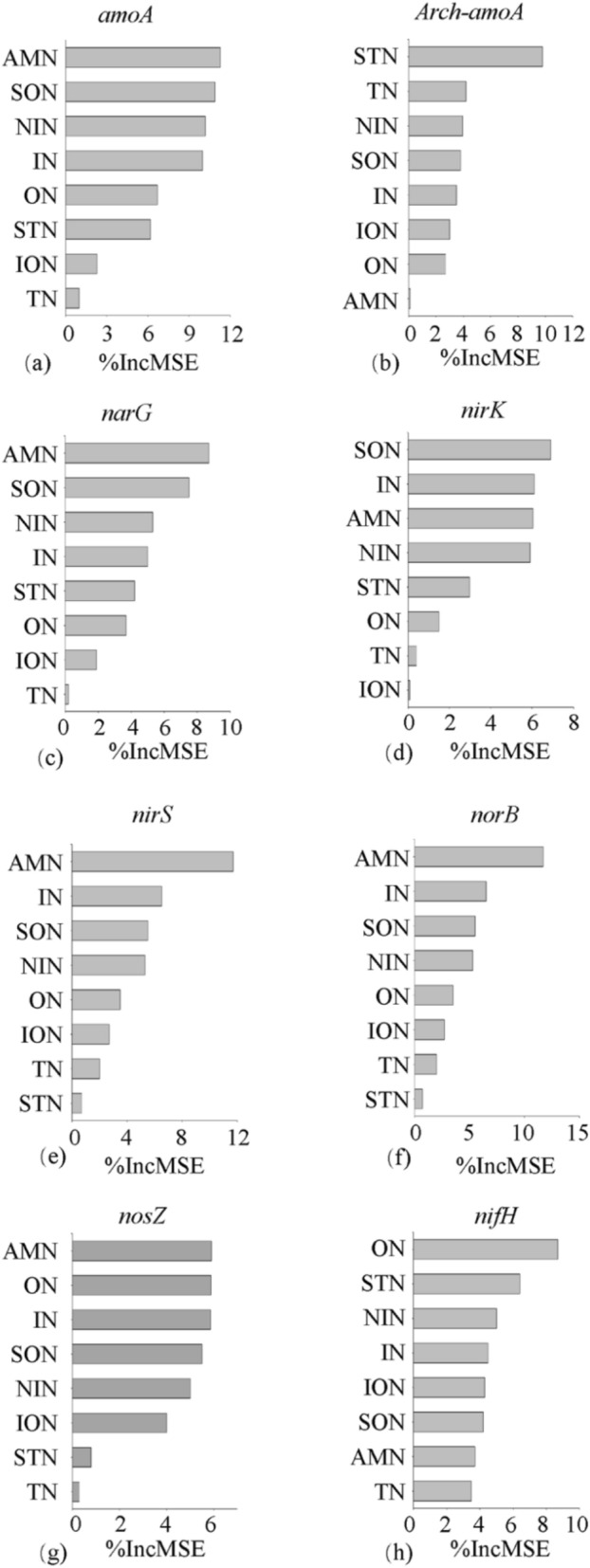
The ABT of the effects of soil nitrogen structure on different gene expression levels.

The relative influence of the different nitrogen forms on *amoA, arch-amoA, narG, nirK, nirS, norB, nosZ* and *nifH* gene copies was determined by the ABT model (Fig. 5), which showed AMN (accounting for 11.3%, 8.7%, 11.7%, 11.8% and 5.9% of the variation) as the major factors determining the abundance of *amoA, narG, nirS, norB* and *nosZ* genes, respectively. STN (9.8%) as the major factors determining the abundance of *Arch-amoA* gene. SON (6.9%) as the major factors determining the abundance of *nirK* gene. ON (8.7%) as the major factors determining the abundance of *nifH* gene.

## Discussion

### Nitrogen forms in tobacco-growing soil were changed by different previous crops

Prolonged tobacco cultivation could lead to soil continuous cropping sickness, and crop rotation was the most straightforward and effective method to break these issues. When tobacco was rotated with different crops, the effects on soil improvement vary. Tobacco, as a crop sensitive to continuous cropping, faced significant continuous cropping obstacles in the Yunnan region of China. The results of this study suggested that two planting methods, barley-tobacco and rapeseed-tobacco, significantly increased the content of ammonium nitrogen and soluble organic nitrogen in tobacco-growing soils compared to continuous tobacco planting. Simultaneously, due to the high nitrogen demand of rapeseed and barley during their growth and development stages, the content of nitrate nitrogen and inorganic nitrogen significantly decreased. Therefore, the cultivation of rapeseed and barley as preceding crops altered the proportion of ammonium nitrogen to nitrate nitrogen in tobacco-growing soils. This proportion, in turn, would have a significant impact on the growth and development of subsequent tobacco crops, as suggested by Ghiasi et al., who demonstrated that the ratio of ammonium nitrogen to nitrate nitrogen in tobacco-growing soils affected the metabolic products of tobacco leaves^26^. Furthermore, the study's results indicated that crop rotation patterned with other crops significantly differ in soil nitrogen structure compared to continuous tobacco planting. While the differences in soil nitrogen structure between barley-tobacco and rapeseed-tobacco planting methods were not pronounced, distinctions still existed in specific nitrogen forms. Jiang et al., demonstrated through a 12-year-long-term trial that crop rotation and continuous cropping altered soil microbial diversity, microbial network stability, biomass, nutrient pools, and microbial resources^27^. Menefee et al., found in their study on various grass cropping systems that the soil health score and microbial phospholipid fatty acids (PLFAs) were the lowest in continuous cropping systems^28^. In the context of tobacco research, Wang et al., discovered in their study on mushroom-tobacco rotation that this rotation altered the nutrient structure in soil and also affected the taxonomic groups and dominant species of bacteria and fungi in the soil^29^. Therefore, we concluded that different preceding crops lead to alterations in the nitrogen structure of tobacco-growing soils. Simultaneously, changes in soil microorganisms in tobacco-growing soils might be a contributing factor to the alterations in soil nitrogen structure.

### Effects of different preceding crops on the expression of nitrogen cycling-related genes in tobacco-growing soil

Continuous tobacco cultivation not only leaded to nutrient imbalances in tobacco-growing soils but also disrupted soil microbial ecosystems. Soil nitrogen cycling is a critical component of soil microbial substance cycling and energy conversion, involving various complex and extensive microorganisms. This study investigated the nitrogen fixation process (N_2_ → NH_3_), nitrification process (NH_4_^+^ → NO_2_^−^ → NO_3_^−^), and denitrification process (NO_3_^−^ → NO_2_^−^ → NO → N_2_O → N_2_) in tobacco-growing soil under the influence of different preceding crops. The results of this study demonstrated that the choice of different preceding crops leaded to changes in the expression of nitrogen cycling-related genes in tobacco-growing soil. Previous research, such as Iannetta et al., had indicated that crop rotation between legumes and non-legumes could enhance soil nitrogen fixation, while Ma et al., found that wheat and rice rotation could reduce the use of nitrogen fertilizers^30,31^. These studies had collectively shown that crop rotation could improve soil nitrogen fixation capacity. However, in this experiment, the rotation of rapeseed and barley instead decreased the expression of the *nifH* gene in the soil, thereby reducing the conversion of N_2_ to NH_3_. This might be related to the pH of the tobacco-growing soil, and Collavino showed that nifH gene abundance, determined by quantitative real-time polymerase chain reaction, was higher in agricultural soils than in non-agricultural soils, and was influenced by soil chemistry under intensive crop rotation but not under monoculture^32^. This process is carried out by ammonia-oxidizing archaea (AOA) and ammonia-oxidizing bacteria (AOB). The results of this study showed that rapeseed and barley, as preceding crops, did not significantly alter the expression of *arch-amoA* in tobacco-growing soils. In contrast, the *amoA* gene was significantly inhibited, with a more pronounced inhibitory effect observed when rapeseed was used as the preceding crop. Simultaneously, rapeseed and barley as preceding crops reduced nitrous oxide emission by limiting the abundance of *nirS* and *nirK* genes. Research by Paungfoo-Lonhienne et al., on legume and sugarcane rotations also indicated that legume crop rotation suppressed the nitrifying microbial community in sugarcane-cropping soils^33^. This experiment's findings suggested that different preceding crops altered the expression of nitrogen cycling-related genes in tobacco-growing soil. This change in gene expression, affecting key genes in the nitrification and denitrification processes, subsequently altered the ratio of ammonium nitrogen to nitrate nitrogen in the soil, making it more conducive to tobacco growth.

### Impact of soil nitrogen structure on the expression of nitrogen cycling-related genes in soil

Changes in soil nutrient cycling-related genes during soil nutrient cycling processes can be influenced by multiple factors. Research conducted by Lee et al., on forest soils demonstrated that *nifH-harboring* bacterial communities tended to assemble along environmental gradients^34^. In another study by Hu et al., both gene and transcript abundanced of *amoA* and *nosZ* were found to be positively correlated with soil nitrous oxide (N_2_O) emissions^35^. In our study, we hypothesized that the composition of nitrogen structures in soil, especially in tobacco-growing soils, could influence the expression of nitrogen cycling-related functional genes. The results of this study indicated that *Arch-amoA* showed no significant correlation with most forms of nitrogen but is significantly negatively correlated with soil total nitrogen. Furthermore, the expression of *amoA* was also inhibited by most nitrogen forms, suggesting that an increase in nitrogen content acted as an environmental factor that inhibited the conversion of NH_4_^+^ to NO_2_^−^. Inorganic nitrogen and nitrate nitrogen inhibited the expression of *narG*, reducing the conversion of NO_3_^−^ to NO_2_^−^ and limiting denitrification. Additionally, inorganic nitrogen and nitrate nitrogen significantly increased the expression of *nirK, nirS, norB*, and *nosZ* genes in the soil, promoting the latter stages of denitrification. Research by Mocniak et al., had shown that nitrate nitrogen was not conducive to the synthesis or accumulation of total sugars, reducing sugars, total nitrogen, and chloride ion content in cured tobacco leaves^36^. Therefore, reducing nitrate nitrogen content in tobacco-growing soil could enhance tobacco flavor. This study also found that ammonium nitrogen strongly influenced the expression of most soil nitrogen cycling functional genes. Therefore, when selecting preceding crops for rotation in tobacco-growing soil, it is advisable to choose crops with an appropriate ratio of ammonium nitrogen to nitrate nitrogen in the soil after the preceding crop's harvest.

## Conclusion

In this study, barley, rapeseed and non-cultivated crops were used as precursor crops to study the nitrogen structure of tobacco-planting soil and the expression of soil nitrogen cycle related genes. The results showed that different previous crops had different effects on the nitrogen content of different forms in the soil of tobacco cultivation. NH_4_^+^-N and NO_3_^−^-N were the two nitrogen forms most affected by the previous crop. The results of this study showed that different previous crops changed the expression levels of nitrogen cycling-related genes in the soil of tobacco cultivation, thus affecting the proportion of various nitrogen forms in the soil. Therefore, when selecting preceding crops for rotation in tobacco cultivation soil, it is advisable to choose crops that result in an appropriate ratio of ammonium nitrogen to nitrate nitrogen in the soil after the preceding crop's harvest.

## Data Availability

The original contributions presented in the study are included in the article, further inquiries can be directed to the corresponding authors.
